# Synthesis, Spectroscopic and Physicochemical Characterization and Biological Activity of Co(II) and Ni(II) Coordination Compounds with 4-Aminoantipyrine Thiosemicarbazone

**DOI:** 10.1155/BCA.2005.271

**Published:** 2005

**Authors:** Ram K. Agarwal, Surendra Prasad

**Affiliations:** Department of Chemistry School of Pure and Applied Sciences The University of the South Pacific Post Box 1168 Suva Fiji

**Keywords:** Cobalt(II), Nickel(II), Complexes, Thiosemicarbazone, Biological Activity

## Abstract

We describe the synthesis and characterization of cobalt(II) and nickel(II) coordination compounds of 4[N-(furan-2’-aldimine)amino]antipyrine thiosemicarbazone (FFAAPTS) and 4[N-(4'-nitrobenzalidene) amino]antipyrine thiosemicarbazone (4'-NO_2_BAAPTS). All the isolated compounds have the general composition MX_2_(L)(H_2_O) (M = Co^2+^ or Ni^2+^; X = Cl, Br, NO_3_, NCS or CH_3_COO; L = FFAAPTS or 4'-NO_2_BAAPTS) and M(ClO_4_)_2_(L)_2_ (M = Co^2+^ or Ni^2+^; L = FFAAPTS or 4'-NO_2_BAAPTS). Infrared spectral studies indicate that both the thiosemicarbazones coordinate in their neutral form and they act as {N,N,S} tridentate chelating ligands. Room temperature magnetic measurements and electronic spectral studies suggest the distorted octahedral geometries of the prepared complexes. Thermogravimetric studies are also reported and the possible structures of the complexes are proposed. Antibacterial and antifungal properties of these metal-coordination compounds have also been studied.


### Synthesis, Spectroscopic and Physicochemical Characterization and Biological Activity of Co(II) and Ni(II) Coordination Compounds with 4-Aminoantipyrine Thiosemicarbazone

Ram K. Agarwal,* and Surendra Prasad*

*Department of Chemistry, School of Pure and Applied Sciences,*
*The University of the South Pacific, Post Box 1168*
*Suva, Fiji Islands,*
*e. mail: ram-agarwal54@yahoo.com,*
*prasad_su@usp.ac.fj*

### ABSTRACT

We describe the synthesis and characterization of cobalt(II) and nickel(II) coordination compounds of 4[N-(furan-2'-aldimine)amino]antipyrine thiosemicarbazone (FFAAPTS) and 4[N-(4'-nitro-benzalidene)amino]antipyrine thiosemicarbazone (4'-$NO_2$BAAPTS). All the isolated compounds have the general composition $MX_2(L)(H_2O)$ (M = $Co^{2+}$ or $Ni^{2+}$; X = Cl, Br, $NO_3$, NCS or $CH_3COO$; L = FFAAPTS or 4'-$NO_2$BAAPTS) and $M(ClO_4)_2(L)_2$ (M = $Co^{2+}$ or $Ni^{2+}$; L = FFAAPTS or 4'-$NO_2$BAAPTS). Infrared spectral studies indicate that both the thiosemicarbazones coordinate in their neutral form and they act as {N,N,S} tridentate chelating ligands. Room temperature magnetic measurements and electronic spectral studies suggest the distorted octahedral geometries of the prepared complexes. Thermogravimetric studies are also reported and the possible structures of the complexes are proposed. Antibacterial and antifungal properties of these metal-coordination compounds have also been studied.

**Key Words:** Cobalt(II), Nickel(II), Complexes, Thiosemicarbazone, Biological Activity.

### INTRODUCTION

Of the sulfur donor ligands, thiosemicarbazones have perhaps not been given as much attention as dithiophosphate /1/, dithiocarbamates /2,3/, dithiolates /4/, dithio-β-diketonates /5/, dithiooxamide /6/ or

---

* Authors for correspondence.

xanthates /3/. Although many thiosemicarbazones possess a wide spectrum of medicinal properties, including activity against influenza /7/, protozoa /8/, smallpox /9/, certain kinds of tumor /10/, tuberculosis /11/, leprosy /12/, bacterial /13/ and viral infections /14/, psoriasis /15/, rheumatism and tripamosomiasis /16/, cocidiosis /17/, malaria /18/, and have been suggested as possible pesticides /19/ and fungicides /20/, their activity has frequently been thought to be due to their ability to chelate trace metals. Liebermeister /21/ showed that copper ions enhance the antitubercular activity of *p*-acetamidobenzaldehyde thiosemicarbazone. Similarly, Petering *et al.* /22/ showed that the active intermediate in the antitumour activity of 3-ethoxy-2-oxobutyraldehyde bis-(thiosemicarbazone) ($H_2KTS$) was the chelate Cu(KTS). These findings have continuously led to an increased interest in the chemistry of metal chelates of thiosemicarbazones. A number of review articles /23-28/ have appeared on various metal-coordination complexes of thiosemicarbazones. In recent years a number of workers have also reported some transition metal coordination complexes of thiosemicarbazones. /29-36/. Comparatively less is known about transition metal coordination complexes of thiosemicarbazones having a pyrazolone ring. In view of this, in the present work we describe the synthesis, spectral, magnetic, thermal and biological properties of $Co^{2+}$ and $Ni^{2+}$ complexes of 4 [N-(furan-2'-aldimine)amino]antipyrine thiosemicarbazone (FFAAPTS) (I) and 4[N-(4'-nitrobenz-alidene)amino]antipyrine thiosemicarbazone (4'-$NO_2$BAAPTS) (II).

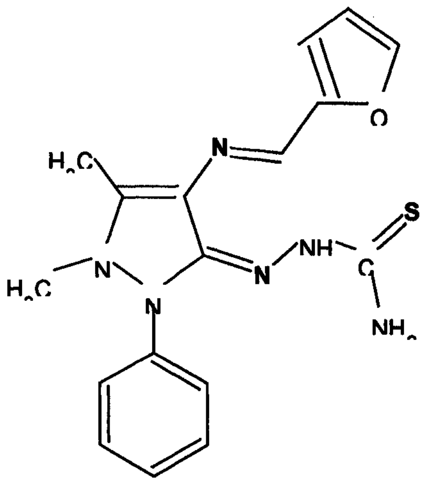

FFAAPTS (I)

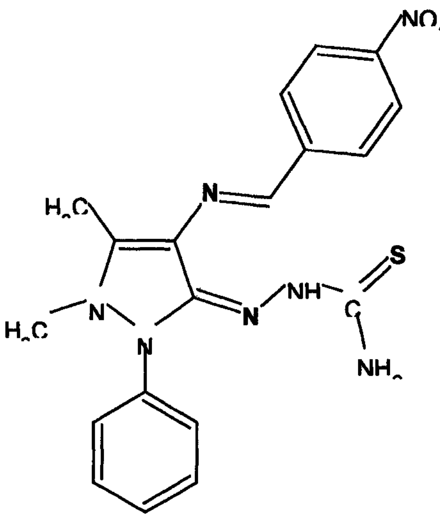

4'-$NO_2$BAAPTS (II)

### EXPERIMENTAL

$MX_2.nH_2O$ (M = $Co^{2+}$ or $Ni^{2+}$; X = Cl, Br, $NO_3$ or $CH_3COO$) were obtained from BDH and were used as such. $M(NCS)_2$ (M = $Co^{2+}$ or $Ni^{2+}$) were prepared by mixing metal chloride (in ethanol) and ethanolic solution of potassium thiocyanate in 1:2 molar ratio. Precipitated KCl was filtered off and filtrate having respective metal thiocyanate was used immediately for complex formation. $M(ClO_4)_2$ (M = $Co^{2+}$ or $Ni^{2+}$)

were prepared by the addition of an ethanolic solution of sodium perchlorate into respective metal chloride solutions. White precipitate of NaCl was filtered off and the filtrate containing $M(ClO_4)_2$ was used as such for complex formation. Both the thiosemicarbazones ligands, *i.e.* FFAAPTS and 4'-$NO_2$BAAPTS, were synthesized in the laboratory as described previously /37/.

### Synthesis of the complexes

### *$Co^{2+}$ complexes of FFAAPTS or 4'-$NO_2$BAAPTS*

All the complexes were prepared by mixing ethanolic solution of ligand and metal salts in required molar ratio (1:1/1:2 where both the ligands were in 10% excess). The reaction mixture was refluxed on a water bath for 2-3 hrs and then concentrated to a small volume on a hot plate at ~50°C. After cooling, desired crystals of the complexes obtained were filtered, recrystallized and washed in the ethanol and dried in vacuum over $P_4O_{10}$.

### *$Ni^{2+}$ complexes of FFAAPTS or 4'-$NO_2$BAAPTS*

A general method was used for the preparation of the complexes. A hot ethanolic solution of the corresponding nickel(II) salt was mixed with a hot ethanolic solution of the respective ligand (in 1:1 or 1:2 molar ratios where both the ligands were in 10% excess). The reaction mixture was refluxed on a water bath for ~2 hrs. On cooling at room temperature, the desired complexes were precipitated out in each case. They were filtered, recrystallized, washed with ethanol and diethyl-ether and finally dried over $P_4O_{10}$ under vacuum.

### Physical measurements and analytical estimations

The cobalt(II) and nickel(II) in their metal complexes were estimated complexometrically with EDTA using murexide and erichrome black-T as indicators respectively after decomposing the complexes with conc. $H_2SO_4$ and $H_2O_2$ /38/. The halogens and pseudo halogens were estimated by Volhard's method /39/. The perchlorate was estimated by the method suggested by Kurz *et al.* /40/. The percentage of sulfur was estimated gravimetrically as $BaSO_4$. The nitrogen content was determined by the Kjeldahl method.

The molecular weight of the complexes was determined in the laboratory cryoscopically in freezing nitrobenzene using a Beckmann thermometer of ±0.01°C accuracy. The conductivity measurements were carried out, at room temperature in nitrobenzene, using a conductivity bridge and dip type cell operated at 220 volts A.C. mains. The magnetic measurements on powder form of the complexes were carried out at room temperature on Evans's balance using anhydrous copper(II) sulfate as calibrant. The infrared spectra of the complexes were recorded on a Perkin Elmer infrared spectrophotometer model Spectrum 1000 in CsI pellets in the range of 4000-200 $cm^{-1}$. Diffused reflectance spectra of the solid compounds were recorded on a Beckmann DK-2A spectrophotometer at C.D.R.I, Lucknow, India. Thermogravimetric studies of the complexes were carried out on Santon Red Craft Thermobalance Model TG-750 in static air with open sample holder and a small boat, the heating rate was 6°C/min.

**RESULTS AND DISCUSSION**

The reaction of $Co^{2+}$ and $Ni^{2+}$ with FFAAPTS and 4'-$NO_2$BAAPTS yielded the $MX_2$(L) ($H_2O$); [M = $Co^{2+}$ or $Ni^{2+}$; X = Cl, Br, $NO_3$, NCS or $CH_3COO$] and $M(ClO_4)_2(L)_2$ [M = $Co^{2+}$ or $Ni^{2+}$; L = FFAAPTS or 4'-$NO_2$BAAPTS]. The analytical data of these complexes showed that the solids are stable and can be stored for months without any significant change in their formulae. These complexes are generally soluble in common organic solvents. The molar conductance values of the complexes in nitrobenzene reveal that the halo, nitrato, isothiocyanato and acetato complexes are essentially non-electrolytes, while the perchlorato complexes dissociate in nitrobenzene and behave as 1:2 electrolytes /41/. The cryoscopic molecular weights and conductivity data are presented in Tables 1 and 2. The molecular weight results are in broad agreement with the conductance data suggesting monomeric formulations.

### Magnetic susceptibility

The magnetic measurements of the cobalt(II) complexes (4.8-5.4 BM) (Table 1) show that all are paramagnetic and have three unpaired electrons indicating a high-spin octahedral configuration. The paramagnetism observed for the present series of $Ni^{2+}$ complexes ranges from 2.6-3.2 BM (Table-2), which is consistent with the octahedral stereochemistry of these complexes.

### Infrared spectra

A study and comparison of infrared spectra of both thiosemicarbazone ligands (FFAAPTS and 4'-$NO_2$BAAPTS) and their $Co^{2+}$ and $Ni^{2+}$ complexes (Tables 3-6) imply that these ligands behave as neutral tridentate and the metals are coordinated through N and N of two azomethine groups and of S of thio-keto group. The strong bands observed at 3440-3270 $cm^{-1}$ region in both thiosemicarbazones have been observed due to N–H vibrations. Practically no effect on these frequencies after complexation preclude the possibility of complexation at this group. The absorptions at ~1600 $cm^{-1}$ in free ligands may be attributed to C=N stretching vibrations of imine-nitrogen which is in agreement with the previous observations[42,43]. On complexation FFAAPTS and 4'-$NO_2$BAAPTS with $Co^{2+}$ and $Ni^{2+}$ these frequencies are shifted to lower energies (Tables 3-6). These observations suggest involvement of unsaturated nitrogen atoms of the two azomethine groups in bonding with the metal ions.

In substituted thioureas, the C=S stretching vibrations contributed greatly to some other vibrations as C–N stretching and bending as well as N–C–S bending modes /44/. In the spectra of the present ligands, the bands observed in the region 1300-1125 $cm^{-1}$, 1125-1095 $cm^{-1}$ and 840-730 $cm^{-1}$ regions are assigned to [ν(C=S)+ν(C=N)+ν(C–N)], δ(N–C–S) + δ(C=S) bending and ν(C=S) stretching, respectively, which are in line with the observations of previous researchers /45,46/. Coordination of sulfur with the metal ions results in the displacement of electrons towards the latter, thus resulting in the weakening of C=S bond. Hence on complexation C=S stretching vibrations should decrease and those of C–N should increase. In all the present complexes of $Co^{2+}$ and $Ni^{2+}$ with FFAAPTS and 4'-$NO_2$BAAPTS, frequencies in the range 1300-1125 $cm^{-1}$

**Table 1**

Analytical, conductivity, molecular weight and magnetic moment data of $Co^{2+}$ complexes of FFAAPTS and 4'-$NO_2$BAAPTS.

| Complex | Yield (%) | Analysis: found (calcd.) % | | | | m.w. Found (Calcd.) | Ωm (ohm$^{-1}$ cm$^2$ mole$^{-1}$) | $\mu_{eff}$ (B.M.) |
|---|---|---|---|---|---|---|---|---|
| | | Co | N | S | Anion | | | |
| $CoCl_2(H_2O)$(FFAAPTS) | 72 | 11.70 (11.75) | 16.63 (16.73) | 6.30 (6.37) | 14.03 (14.14) | 496 (502) | 3.1 | 3.1 |
| $CoBr_2(H_2O)$(FFAAPTS) | 70 | 9.92 (9.98) | 14.10 (14.21) | 5.32 (5.41) | 26.97 (27.07) | 587 (591) | 3.7 | 2.9 |
| $Co(NO_3)_2(H_2O)$(FFAAPTS) | 75 | 10.57 (10.63) | 20.06 (20.18) | 5.70 (5.76) | - | 551 (555) | 3.9 | 3.2 |
| $Co(NCS)_2(H_2O)$(FFAAPTS) | 70 | 10.70 (10.78) | 20.33 (20.47) | 17.49 (17.55) | 21.04 (21.20) | 544 (547) | 4.0 | 2.6 |
| $Co(CH_3COO)_2(H_2O)$(FFAAPTS) | 68 | 10.67 (10.74) | 15.20 (15.30) | 5.76 (5.82) | - | 546 (549) | 3.4 | 3.2 |
| $Co(ClO_4)_2(FFAAPTS)_2$ | 70 | 6.05 (6.10) | 17.27 (17.39) | 6.55 (6.62) | 20.50 (20.60) | 318 (966) | 52.3 | 3.0 |
| $CoCl_2(H_2O)$(4'-$NO_2$BAAPTS) | 70 | 10.52 (10.59) | 17.46 (17.59) | 5.69 (5.74) | 12.63 (12.74) | 553 (557) | 3.7 | 5.1 |
| $CoBr_2(H_2O)$(4'-$NO_2$BAAPTS) | 70 | 9.06 (9.13) | 15.08 (15.17) | 4.91 (4.95) | 24.63 (24.76) | 641 (646) | 3.4 | 4.9 |
| $Co(NO_3)_2(H_2O)$(4'-$NO_2$BAAPTS) | 72 | 9.61 (9.67) | 20.79 (20.93) | 5.17 (5.24) | - | 607 (610) | 2.7 | 5.0 |
| $Co(NCS)_2(H_2O)$(4'-$NO_2$BAAPTS) | 65 | 9.72 (9.80) | 20.50 (20.65) | 15.83 (15.94) | 19.17 (19.26) | 597 (602) | 3.1 | 4.8 |
| $Co(CH_3COO)_2(H_2O)$(4'$NO_2$BAAPTS) | 60 | 9.69 (9.76) | 16.14 (16.22) | 5.23 (5.29) | - | 599 (604) | 2.9 | 5.2 |
| $Co(ClO_4)_2$(4'-$NO_2$BAAPTS)$_2$ | 60 | 5.40 (5.48) | 18.13 (18.21) | 5.87 (5.94) | 18.30 (18.49) | 356 (1076) | 51.9 | 5.4 |

**Table 2**

Analytical, conductivity, molecular weight and magnetic moment data of $Ni^{2+}$ complexes of FFAAPTS and 4'-$NO_2$BAAPTS.

| Complex | Yield (%) | Analysis : Found (Calcd.) % | | | | m.w. Found (Calcd.) | Ωm ($ohm^{-1}$ $cm^2$ $mole^{-1}$) | $\mu_{eff}$ (B.M.) |
|---|---|---|---|---|---|---|---|---|
| | | Ni | N | S | Anion | | | |
| $NiCl_2(H_2O)$(FFAAPTS) | 75 | 11.71 (11.75) | 16.62 (16.73) | 6.29 (6.37) | 14.04 (14.14) | 497 (502) | 3.1 | 3.1 |
| $NiBr_2(H_2O)$(FFAAPTS) | 70 | 9.90 (9.98) | 14.10 (14.21) | 5.32 (5.41) | 26.95 (27.07) | 597 (591) | 3.7 | 2.9 |
| $Ni(NO_3)_2(H_2O)$(FFAAPTS) | 75 | 10.55 (10.63) | 20.06 (20.18) | 5.70 (5.76) | - | 551 (555) | 3.9 | 3.2 |
| $Ni(NCS)_2(H_2O)$(FFAAPTS) | 72 | 10.69 (10.78) | 20.35 (20.47) | 17.47 (17.55) | 21.06 (21.20) | 543 (547) | 4.0 | 2.6 |
| $Ni(CH_3COO)_2(H_2O)$(FFAAPTS) | 70 | 10.64 (10.74) | 15.19 (15.30) | 6.76 (5.82) | - | 545 (549) | 3.4 | 3.2 |
| $Ni(ClO_4)_2(FFAAPTS)_2$ | 70 | 6.03 (6.10) | 17.26 (17.39) | 6.55 (6.62) | 20.48 (20.60) | 319 (966) | 52.3 | 3.0 |
| $NiCl_2(H_2O)$(4'-$NO_2$BAAPTS) | 70 | 10.51 (10.59) | 17.47 (17.59) | 5.68 (5.74) | 12.63 (12.74) | 553 (557) | 3.9 | 2.9 |
| $NiBr_2(H_2O)$(4'-$NO_2$BAAPTS) | 68 | 9.06 (9.13) | 15.07 (15.17) | 4.90 (4.95) | 24.64 (24.76) | 642 (646) | 4.1 | 3.0 |
| $Ni(NO_3)_2(H_2O)$(4'-$NO_2$BAAPTS) | 72 | 9.62 (9.67) | 20.80 (20.93) | 5.18 (5.24) | - | 606 (610) | 2.9 | 3.2 |
| $Ni(NCS)_2(H_2O)$(4'-$NO_2$BAAPTS) | 70 | 9.71 (9.80) | 20.52 (20.65) | 15.82 (15.94) | 19.15 (19.26) | 598 (602) | 3.6 | 2.8 |
| $Ni(CH_3COO)_2(H_2O)$(4'-$NO_2$BAAPTS) | 70 | 9.67 (9.76) | 16.13 (16.22) | 5.23 (5.29) | - | 599 (604) | 3.9 | 2.7 |
| $Ni(ClO_4)_2$(4'-$NO_2$BAAPTS$)_2$ | 65 | 5.41 (5.48) | 18.13 (18.21) | 5.85 (5.94) | 18.32 (18.49) | 355 (1076) | 52.9 | 2.9 |

**Table 3**

Infrared Absorption frequencies ($cm^{-1}$) of $Co^{2+}$ complexes of FFAAPTS.

| Assignments | FFAAPTS | $CoCl_2$ (FFAAPTS). $H_2O$ | $CoBr_2$ (FFAAPTS). $H_2O$ | $Co(NO_3)_2$ (FFAAPTS). $H_2O$ | $Co(NCS)_2$ (FFAAPTS). $H_2O$ | $Co(CH_3COO)_2$ (FFAAPTS). $H_2O$ | $Co(ClO_4)_2$ $(FFAAPTS)_2$ |
|---|---|---|---|---|---|---|---|
| ν(N–H) | 3440 s<br>3280 s | 3442 s<br>3280 m | 3440 s<br>3282 m | 3445 s<br>3282 m | 3442 s<br>3285 m | 3440 s<br>3282 m | 3442 m<br>3280 m |
| ν(C=N) | 1600 s | 1570 s | 1550 s | 1550 s | 1542 s | 1552 s | 1550 m |
| ν(C=S) +<br>ν(C=N) +<br>ν(C–N) | 1315 s<br>1185 m | 1350 s<br>1220 m | 1335 m<br>1210 m | 1330 m<br>1215 m | 1332 m<br>1220 m | 1330 m<br>1222 m | 1335 m<br>1215 m |
| δ(N–C–S) +<br>C–S bending | 1122 m<br>1095 m | 1162 m<br>1110 m | 1155 m<br>1112 m | 1150 m<br>1115 m | 1152 m<br>1112 m | 1155 m<br>1115 m | 1150 m<br>1120 m |
| ν(N–N) | 1040 m | 1052 m | 1055 m | 1058 m | 1050 m | 1052 m | 1055 m |
| ν(C=S) | 840 s<br>820 m<br>780 s | 810 m<br>795 m | 812 m<br>790 m | 815 m<br>792 m | 825 m<br>795 m | 815 m<br>775 m | 812 m<br>780 m |
| ν(Co–N)<br>ν(Co–S) | - | 410 m<br>345 w | 405 m<br>340 w | 412 m<br>342 w | 408 m<br>340 w | 410 m<br>350 w | 405 m<br>345 w |
| ν(Co–Cl) | - | 342 w | - | - | - | - | - |

**Table 4**

Infrared absorption frequencies ($cm^{-1}$) of $Co^{2+}$ complexes of 4'-$NO_2$BAAPTS.

| Assignments | 4'-$NO_2$BAAPTS | $CoCl_2$ 4'-($NO_2$BAAPTS). $H_2O$ | $CoBr_2$ (4'-$NO_2$BAAPTS). $H_2O$ | $Co(NO_3)_2$ (4'-BAAPTS). $H_2O$ | $Co(NCS)_2$ (4'-$NO_2$BAAPTS). $H_2O$ | $Co(CH_3COO)_2$ (4'-$NO_2$BAAPTS). $H_2O$ | $Co(ClO_4)_2$ 2(4'-$NO_2$BAAPTS). |
|---|---|---|---|---|---|---|---|
| ν(N–H) | 3355 s<br>3330 m | 3350 m<br>3330 m | 3358 s<br>3335 w | 3360 m<br>3332 m | 3360 s<br>3332 m | 3358 m<br>3330 m | 3360 m<br>3332 w |
| ν(C=N) | 1600 s | 1578 m | 1575 m | 1565 m | 1570 m | 1565 m | 1570 m |
| ν(C=S) +<br>ν(C=N) +<br>ν(C–N) | 1310 m<br>1290 m | 1360 m<br>1315 m | 1370 m<br>1320 m | 1375 m<br>1322 m | 1370 m<br>1340 m | 1372 m<br>1340 m | 1365 m<br>1335 m |
| δ(N–C–S) +<br>C–S bending | 1115 m<br>1095 w | 1150 m<br>1130 m | 1160 m<br>1130 m | 1162 m<br>1138 m | 1165 m<br>1142 m | 1155 m<br>1135 m | 1160 m<br>1135 m |
| ν(N–N) | 10 52 m | 1062 m | 1065 m | 1062 m | 1065 m | 1068 m | 1065 m |
| ν(C=S) | 832 s<br>730 m | 780 s<br>740 m | 770 s<br>745 m | 755 s<br>735 m | 772 s<br>745 m | 770 m<br>742 m | 765 s<br>732 m |
| ν(Co–N)<br>ν(Co–S) | - | 410 m<br>342 w | 410 m<br>332 w | 405 m<br>330 w | 412 m<br>332 w | 410 m<br>340 w | 408 m<br>335 w |
| ν(Co–Cl) | - | 325 w | - | - | - | - | - |

**Table 5**

Infrared Absorption frequencies ($cm^{-1}$) of $Ni^{2+}$ complexes of FFAAPTS.

| Assignments | FFAAPTS | $NiCl_2$ (FFAAPTS). $H_2O$ | $NiBr_2$ (FFAAPTS). $H_2O$ | $Ni(NO_3)_2$ (FFAAPTS). $H_2O$ | $Ni(NCS)_2$ (FFAAPTS). $H_2O$) | $Ni(CH_3COO)_2$ (FFAAPTS). $H_2O$ | $Ni(ClO_4)_2$ $(FFAAPTS)_2$ |
|---|---|---|---|---|---|---|---|
| ν(N–H) | 3440 s<br>3280 s | 3445 s<br>3280 m | 3442 s<br>3282 m | 3445 s<br>3285 m | 3442 s<br>3280 m | 3442 m<br>3282 m | 3440 s<br>3282 m |
| ν(C=N) | 1600 s | 1552 s | 1565 s | 1555 s | 1560 s | 1562 s | 1555 s |
| ν(C=S) + ν(C=N) + ν(C–N) | 1315 s<br>1185 m | 1330 s<br>1216 m | 1350 s<br>1220 m | 1335 m<br>1210 m | 1330 m<br>1215 m | 1332 m<br>1222 m | 1335 m<br>1215 m |
| δ(N–C–S)+ C–S bending | 1122 m<br>1095 m | 1152 m<br>1115 m | 1162 m<br>1112 m | 1155 m<br>1115 m | 1152 m<br>1112 m | 1155 m<br>1115 m | 1150 m<br>1122 m |
| ν(N–N) | 1040 m | 1058 m | 1055 m | 1060 m | 1058 m | 1055 m | 1052 m |
| ν(C=S) | 840 s<br>820 m<br>780 s | 820 m<br>795 m | 815 m<br>792 m | 825 m<br>795 m | 815 m<br>775 m | 812 m<br>782 m | 812 m<br>792 m |
| ν(Ni–N)/ ν(Ni–S) | - | 410 m<br>345 w | 405 m<br>352 w | 408 m<br>348 w | 412 m<br>345 w | 408 m<br>348 w | 410 m<br>345 w |
| ν(Ni–Cl) | - | 345 w | - | - | - | - | - |

**Table 6**

Infrared Absorption frequencies ($cm^{-1}$) of $Ni^{2+}$ complexes of 4'-$NO_2$BAAPTS.

| Assignments | 4'-$NO_2$BAAPTS | $NiCl_2$ 4'-($NO_2$BAAPTS). $H_2O$ | $NiBr_2$ (4'-$NO_2$BAAPTS). $H_2O$ | $Ni(NO_3)_2$ (4'-$NO_2$BAAPTS). $H_2O$ | $Ni(NCS)_2$ (4'-$NO_2$BAAPTS). $H_2O$ | $Ni(CH_3COO)_2$ (4'-$NO_2$BAAPTS). $H_2O$ | $Ni(ClO_4)_2$ $(4'\text{-}NO_2BAAPTS)_2$ |
|---|---|---|---|---|---|---|---|
| ν(N–H) | 3355 s<br>3330 m | 3365 m<br>3332 m | 3360 m<br>3332 w | 3362 m<br>3335 w | 3365 s<br>3330 m | 3358 m<br>3332 m | 3335 m<br>3330 w |
| ν(C=N) | 1600 s | 1568 m | 1570 s | 1565 m | 1550 m | 1545 m | 1552 m |
| ν(C=S)+ ν(C=N)+ ν(C–N) | 1310 m<br>1290 m | 1370 m<br>1340 m | 1373 m<br>1335 m | 1370 m<br>1342 m | 1372 m<br>1335 m | 1378 m<br>1340 m | 1375 m<br>1335 m |
| δ(N–C–S)+ C–S bending | 1115 m<br>1095 w | 1160 m<br>1120 w | 1165 m<br>1130 w | 1168 m<br>1135 w | 1170 m<br>1125 w | 1172 m<br>1128 w | 1168 m<br>1130 w |
| ν(N–N) | 1052 m | 1062 m | 1065 m | 1068 m | 1065 m | 1060 m | 1065 m |
| ν (C=S) | 832 s<br>730 m | 772 s<br>705 m | 775 m<br>715 m | 780 m<br>710 m | 778 m<br>718 m | 780 m<br>712 m | 770 s<br>715 m |
| ν(Ni–N)/ ν(Ni–S) | - | 410 m<br>345 w | 408 m<br>352 w | 412 m<br>340 w | 408 m<br>355 w | 412 m<br>350 w | 410 m<br>348 w |
| ν(Ni–Cl) | - | 342 w | - | - | - | - | - |

increased by 50-60 $cm^{-1}$. Similarly, bending modes of N–C–S and C=S also increased but to a lesser extent. On the other hand, on complexation the frequencies in region 840-730 $cm^{-1}$ were shifted to lower wave numbers and intensity of the bands were also reduced. The changes described are not peculiar and they suggest (C=S) coordination.

The possibility of thione-thiol tautomerism (H–N–C=S $\rightleftharpoons$ C=N–SH) in these ligands has been ruled out for no bands around 2700-2500 $cm^{-1}$, which is characteristic of thiol groups displayed in the infrared absorption /47,48/. In the far infrared spectral region, some new bands with medium to weak intensity in region 410-350 $cm^{-1}$ were assigned to ν(M–N) and ν(M–S). Thus the infrared spectral suggested the tridentate nature of the thiosemicarbazones and pointed out the N,N,S sites as possible donor atoms. In these complexes, the presence of coordinated water was suggested by the very broad absorption centered around 3450 $cm^{-1}$ in their infrared spectra. Bands at ~930 and 770 $cm^{-1}$ may be attributed to rocking and wagging modes of the coordinated water /49,50/.

In all the perchlorato complexes, the presence of the $\nu_3$ (1100-1080 $cm^{-1}$) and $\nu_4$ (625-620 $cm^{-1}$) bands showed that the $T_d$ symmetry of $ClO_4^-$ is maintained in all the complexes. This suggested the presence of $ClO_4^-$ outside the coordination sphere in these complexes /51,52/. In thiocyanato complexes, the three fundamental absorption C–N stretch ($\nu_1$), C–S stretch ($\nu_3$) and N–C–S bending ($\nu_2$) were identified in regions 2045-2030, 845-840 and 475-460 $cm^{-1}$ respectively. These frequencies are associated with the terminal N-bonded isothiocyanate ions /53/.

In nitrato complexes, the infrared data indicated the occurrence of two strong absorption bands in 1560-1520 $cm^{-1}$ and 1310-1300 $cm^{-1}$ regions, which were attributed to $\nu_4$ and $\nu_1$ modes of vibrations of the covalently bonded nitrate groups, respectively /54/. If the ($\nu_4$-$\nu_1$) is taken as an approximate measure of the covalency of nitrate group /55,56/, a value of ~220 $cm^{-1}$ for these complexes suggested strong covalency for the metal-nitrate bonding. Lever *et al.* /57/ have shown that the number and relative energies of nitrate combination frequencies ($\nu_1$+$\nu_4$) in the region 1800-1700 $cm^{-1}$ of the infrared spectrum, may be used as an aid to distinguish the various coordination modes of the nitrato group. Lever *et al.* /57/ have suggested that bidentate coordination of the nitrato group involves a greater distortion from $D_{3h}$ symmetry than unidentate coordination, therefore, bidentate nitrate groups should show a larger separation of ($\nu_1$+$\nu_4$). After an investigation of the spectra of a number of compounds of known crystal structure, Lever *et al.* /57/ showed this to be true; the separation for monodentate nitrate groups appeared to be 5-26 $cm^{-1}$ and that for bidentate groups 25-66 $cm^{-1}$. In the present complexes, a separation of 15-25 $cm^{-1}$ in the combination bands ($\nu_1$+$\nu_4$) in the 1800-1700 $cm^{-1}$ region concluded the monodentate nitrate coordination. In acetato complexes, the $\nu_{asym}(COO^-)$ of free acetate ions are at ~1560 and 1415 $cm^{-1}$ respectively. In the unidentate complex, ν(C=O) is higher than $\nu_{asym}(COO^-)$ and ν(C–O) is lower than $\nu_{sym}(COO^-)$. As a result, the separation between the two ν(C–O) is much larger in unidentate complexes than free ion. The opposite trend is observed in the bidentate complex, *i.e.* the separation between the ν(C–O) is smaller than that of free ion in this case. In the bridging complexes, however, two ν(C–O) are closed to the free ion values. The present complexes showed infrared absorption frequency bands corresponding to $\nu_{asym}(COO^-)$ and $\nu_{sym}(COO^-)$ at ~1610 and 1370 $cm^{-1}$, respectively. These observations indicated that both the acetate groups in the present complexes are unidentate /58,59/.

### Electronic spectra

The electronic spectra of all the $Co^{2+}$ complexes recorded were very similar to each other and consist of two bands in the regions 18500-18000 $cm^{-1}$ and 21000-20000 $cm^{-1}$, which clearly indicated the octahedral stereochemistry of the complexes. The band maxima and their assignments are presented in Table 7. In the present work the ligand field parameters were calculated by the methods given by Reedijk *et al.* /60/ for the ligand field spectra of octahedral $Co^{2+}$ complexes. The energy of $\nu_1$ corresponds to 10 $D_q$ for weak field and the value of Dq is obtained from it. With these assignments, the calculated ligand field parameters B and $D_q$ have also been calculated and given in Table 7. The existence of distortion from a regular octahedral structure was revealed by appreciable intensity enhancement in all the $Co^{2+}$ complexes studied. Apart from this, no differences in the spectra of regular and pseudo octahedral complexes of $Co^{2+}$ were observed.

The absorption spectra of the $Ni^{2+}$ complexes studied displayed bands (Table 8) in 11000-8200 $cm^{-1}$ ($\nu_1$), 17500-15400 $cm^{-1}$ ($\nu_2$) and 27500-24500 $cm^{-1}$ ($\nu_3$) suggested the octahedral stereochemistry of these complexes /61,62/. The calculated 10 $D_q$ values are also included in Table 8. In the corresponding $[NiCl_6]^{2-}$, $[NiBr_6]^{2-}$ or $[Ni(NCS)_6]^{2-}$, the 10 $D_q$ values are of the order of 7200, 7000 and 9700 $cm^{-1}$ respectively. Comparing these values with our results, we can say that there is a weakening effect of axial ligand strength in the complexes. This weakening effect of the axial ligands is expected because the equatorial ligands exert a strong steric hinderance preventing axial ligands from approaching the central metal as closely as would be required for optimum covalent bonding /63/.

### Thermogravimetric studies

The thermogravimetric results of cobalt(II) complexes of FFAAPTS ($L_1$) are presented in Table 9. The thermogravimetric data indicated that the complexes were stable up to 85°C and non-hygroscopic in nature. At temperature range of 80-125°C, one coordinated water molecule is lost, after which decomposition and deligation processes started. Finally, at ~600°C, $Co_3O_4$ was obtained as final residue. The thermal decomposition may be represented by the following equations:

$$[Co(L_1)(H_2O)Cl_2] \xrightarrow{85-125\,^{\circ}C} [Co(L_1)Cl_2] \xrightarrow{200-280\,^{\circ}C}$$

$$[Co(L_1)_{0.5}Cl_2] \xrightarrow{320-360\,^{\circ}C} [CoCl_2] \xrightarrow{520-600\,^{\circ}C} Co_3O_4.$$

$$[Co(L_1)(H_2O)(NO_3)_2] \xrightarrow{80-125\,^{\circ}C} [Co(L_1)(NO_3)_2] \xrightarrow{190-260\,^{\circ}C}$$

$$[Co(L_1)_{0.5}(NO_3)_2] \xrightarrow{320-370\,^{\circ}C} [Co(NO_3)_2] \xrightarrow{530-605\,^{\circ}C} Co_3O_4.$$

$$[Co(L_1)(H_2O)(NCS)_2] \xrightarrow{85-125\,^{\circ}C} [Co(L_1)(NCS)_2] \xrightarrow{180-275\,^{\circ}C}$$

$$[Co(L_1)_{0.5}(NCS)_2] \xrightarrow{315-365\,^{\circ}C} [Co(NCS)_2] \xrightarrow{530-610\,^{\circ}C} Co_3O_4.$$

**Table 7**

Electronic spectral data ($cm^{-1}$) and ligand field parameters of Co(II) complexes of FFAAPTS.

| **Complex** | $\nu_2$ $^4T_{1g}(F)\rightarrow^4A_{2g}$ | $N_3$ $^4T_{1g}(F)\rightarrow^4T_{1g}(P)$ | $D_Q$ ($CM^{-1}$) | B ($cm^{-1}$) | β | $D_q/\beta$ | $\nu_1$ ($cm^{-1}$) |
|---|---|---|---|---|---|---|---|
| $CoCl_2(FFAAPTS).H_2O$ | 18520 | 20000 | 1115 | 1070 | 0.96 | 1.04 | 8770 |
| $CoBr_2(FFAAPTS).H_2O$ | 18180 | 20000 | 1104 | 1060 | 0.95 | 1.04 | 8700 |
| $Co(NO_3)_2(FFAAPTS).H_2O$ | 18000 | 20835 | 1104 | 1060 | 0.95 | 1.04 | 8690 |
| $Co(NCS)_2(FFAAPTS).H_2O$ | 18100 | 20835 | 1105 | 1061 | 0.95 | 1.04 | 8700 |
| $Co(CH_3COO)_2(FFAAPTS).H_2O$ | 18520 | 20000 | 1105 | 1070 | 0.96 | 1.04 | 8772 |
| $Co(ClO_4)_2(FFAAPTS)_2$ | 18000 | 20833 | 1104 | 1060 | 0.95 | 1.04 | 8690 |
| $CoCl_2(4'\text{-}NO_2BAAPTS).H_2O$ | 18000 | 20833 | 1104 | 1060 | 0.95 | 1.04 | 8690 |
| $Co(NO_3)_2(4'\text{-}NO_2BAAPTS).H_2O$ | 18180 | 20000 | 1104 | 1060 | 0.95 | 1.04 | 8700 |
| $Co(NCS)_2(4'\text{-}NO_2BAAPTS).H_2O$ | 18520 | 20835 | 1104 | 1060 | 9,95 | 1.04 | 8696 |
| $Co(CH_3COO)_2(4'\text{-}NO_2BAAPTS).H_2O$ | 18180 | 20000 | 1115 | 1070 | 0.96 | 1.04 | 8772 |
| $Co(ClO_4)_2(4'\text{-}NO_2BAAPTS)_2$ | 18180 | 20835 | 1105 | 1061 | 0.95 | 1.04 | 8700 |

**Table 8**

Electronic spectral data ($cm^{-1}$) and ligand field parameters of Ni(II) complexes of FFAAPTS.

| Complex | $\nu_1$ | $\nu_2$ | $\nu_3$ | $D_q$ ($cm^{-1}$) | B ($cm^{-1}$) | β |
|---|---|---|---|---|---|---|
| $NiCl_2(FFAAPTS).H_2O$ | 9600 | 16200 | 24400 | 960 | 1043 | 0.96 |
| $Ni(NO_3)_2(FFAAPTS).H_2O$ | 9900 | 16600 | 24390 | 990 | 1076 | 0.99 |
| $Ni(NCS)_2(FFAAPTS).H_2O$ | 9800 | 16700 | 24500 | 980 | 1065 | 0.98 |
| $Ni(CH_3COO)_2(FFAAPTS).H_2O$ | 9600 | 15380 | 25640 | 960 | 1093 | 0.96 |
| $Ni(ClO_4)_2(FFAAPTS)_2$ | 9090 | 15150 | 25000 | 910 | 988 | 0.91 |
| $NiCl_2(4'\text{-}NO_2BAAPTS).H_2O$ | 8240<br>10870 | 17540 | 27500 | 1087 | 829 | 0.79 |
| $Ni(NO_3)_2(4'\text{-}NO_2BAAPTS).H_2O$ | 8270<br>10930 | 17700 | 27200 | 1093 | 794 | 0.76 |
| $Ni(NCS)_2(4'\text{-}NO_2BAAPTS).H_2O$ | 8200<br>10810 | 17540 | 26950 | 1081 | 804 | 0.77 |
| $Ni(CH_3COO)_2(4'\text{-}NO_2BAAPTS).H_2O$ | 10990 | 16950 | 27400 | 1099 | 750 | 0.73 |
| $Ni(ClO_4)_2(4'\text{-}NO_2BAAPTS)_2$ | 10930 | 17700 | 27000 | 1093 | 794 | 0.76 |

**Table 9**

Thermoanalytical results obtained for the Co(II) complexes of FFAAPTS.

| Complex | Decomp, Temp. (°C) | | Decomp. Products | Weight loss (%) | |
|---|---|---|---|---|---|
| | Initial | Final | | Found | Calcd. |
| $CoCl_2(FFAAPTS).H_2O$ | 85 | 120 | $CoCl_2(FFAAPTS)$ | 3.66 | 3.58 |
| | 200 | 280 | $CoCl_2(FFAAPTS)_{0.5}$ | 39.98 | 38.84 |
| | 320 | 360 | $CoCl_2$ | 75.20 | 74.10 |
| | 520 | 600 | $Co_3O_4$ | 85.10 | 83.99 |
| $Co(NO_3)_2(FFAAPTS).H_2O$ | 80 | 120 | $Co(NO_3)_2(FFAAPTS)$ | 3.40 | 3.24 |
| | 190 | 260 | $Co(NO_3)_2(FFAAPTS)_{0.5}$ | 36.10 | 35.13 |
| | 320 | 370 | $Co(NO_3)_2$ | 68.00 | 67.02 |
| | 530 | 605 | $Co_3O_4$ | 86.70 | 85.52 |
| $Co(NCS)_2(FFAAPTS).H_2O$ | 85 | 125 | $Co(NCS)_2(FFAAPTS)$ | 3.46 | 3.29 |
| | 180 | 275 | $Co(NCS)_2(FFAAPTS)_{0.5}$ | 36.32 | 35.64 |
| | 315 | 365 | $Co(NCS)_2$ | 68.86 | 68.00 |
| | 530 | 610 | $Co_3O_4$ | 86.20 | 85.31 |
| $NiBr_2(4'\text{-}NO_2BAAPTS).H_2O$ | 80 | 125 | $NiBr_2(4'\text{-}NO_2BAAPTS)$ | 2.86 | 2.78 |
| | 175 | 260 | $NiBr_2(4'\text{-}NO_2BAAPTS)_{0.5}$ | 35.02 | 34.44 |
| | 300 | 360 | $NiBr_2$ | 66.80 | 66.09 |
| | 505 | 590 | NiO | 89.10 | 88.39 |
| $Ni(NO_3)_2(4'\text{-}NO_2BAAPTS).H_2O$ | 85 | 120 | $Ni(NO_3)_2(4'\text{-}NO_2BAAPTS)$ | 3.10 | 2.95 |
| | 190 | 265 | $Ni(NO_3)_2(4'\text{-}NO_2BAAPTS)_{0.5}$ | 37.10 | 36.47 |
| | 300 | 380 | $Ni(NO_3)_2$ | 70.90 | 70.00 |
| | 535 | 610 | NiO | 88.20 | 87.70 |
| $Ni(NCS)_2(4'\text{-}NO_2BAAPTS).H_2O$ | 85 | 125 | $Ni(NCS)_2(4'\text{-}NO_2BAAPTS)$ | 3.10 | 2.99 |
| | 185 | 260 | $Ni(NCS)_2(4'\text{-}NO_2BAAPTS)_{0.5}$ | 37.50 | 36.96 |
| | 280 | 370 | $Ni(NCS)_2$ | 71.80 | 70.93 |
| | 525 | 605 | NiO | 88.30 | 87.54 |

The thermal results of Ni(II) complexes of 4'-$NO_2$BAAPTS ($L_2$) are also presented in Table 9. The careful analysis of thermogravimetric curves showed the following thermal equations:

$$[Ni(L_2)(H_2O)Br_2] \xrightarrow{85-120\,^\circ C} [Ni(L_2)Br_2] \xrightarrow{175-260\,^\circ C}$$

$$[Ni(L_2)_{0.5}Br_2] \xrightarrow{300-360\,^\circ C} [NiBr_2] \xrightarrow{505-590\,^\circ C} NiO$$

$$[Ni(L_2)(H_2O)(NO_3)_2] \xrightarrow{85-120\,^\circ C} [Ni(L_2)(NO_3)_2] \xrightarrow{195-265\,^\circ C}$$

$$[Ni(L_2)_{0.5}(NO_3)_2] \xrightarrow{300-380\,^\circ C} [Ni(NO_3)_2] \xrightarrow{535-610\,^\circ C} NiO$$

$$[Ni(L_2)(H_2O)(NCS)_2] \xrightarrow{85-125\,^\circ C} [Ni(L_2)(NCS)_2] \xrightarrow{195-265\,^\circ C}$$

$$[Ni(L_2)_{0.5}(NCS)_2] \xrightarrow{280-370\,^\circ C} [Ni(NCS)_2] \xrightarrow{525-605\,^\circ C} NiO$$

### Biological properties

A number of workers /27,31,64-67/ were interested in investigating the biological and medicinal properties of transition metal complexes of thiosemicarbazones. Thomas and Parmeswaran /31/ studied the antitumour activities of $Mn^{2+}$, $Co^{2+}$, $Ni^{2+}$ and $Cu^{2+}$ chelates of anthracene-9-carboxaldehyde thiosemicarbazone. Murthy and Dharmaraja /64/ reported the cytotoxic activity of phenylglyoxal bis(thiosemicarbazone) against *Ehrlich ascites* carcinona cells. These compounds were also screened for antimicrobial activity on *B.subtilis* and *E.coli*. They inhibited the bacterial growth considerably. In the present studies, the antibacterial activities of the cobalt(II) complexes and standard drugs (ampicillin and teracycline) were screened by agar-cup method in DMF solvent at a concentration of 50 μg/ml; the results were checked against gram positive bacteria *B.subtilis* and *S.aureus* and gram negative bacteria *E.coli* and *S.typhi* (Table 10). The diameters of zone of inhibition (in mm) of the standard drug ampicillin against gram positive bacteria *B.subtilis* and *S.aureus* and gram negative bacteria *E.coli* and *S.typhi* were found to be 24, 22, 17 and 16 respectively, while tetracycline gave 18, 17, 21 and 22 respectively. Under identical conditions, Table 10 shows that all the cobalt(II)–thiosemicarbazone complexes have moderate antibacterial activities against these bacteria. Both thiosemicarbazones and their cobalt(II) complexes were screened for their antifungal activities against two fungi (*A.niger* and *C.albicans*). The results (Table-10) showed that almost all complexes showed nearly the same extent of activity, but they are less active compared to salicylic acid. It is interesting to note that due to presence of furan ring and comparatively faster diffusion of FFAAPTS complexes showed increased antifungal activity than that of 4'-$NO_2$BAAPTS complexes. These compounds were found to be efficient antifungal agents.

**Table 10**

Antifungal and antibacterial activities of cobalt(II) complexes of FFAAPTS and 4'-$NO_2$BAAPTS.

| **Complex** | **Antibacterial activity** **Zone of inhibition in numbers** | | | | **Antifungal Action** | |
|---|---|---|---|---|---|---|
| | **B. s** | **S.a** | **E.c.** | **S.t** | **A niger** | **albicans**[c] |
| $CoCl_2(H_2O)$(FFAAPTS) | 14 | 15 | 13 | 14 | ++ | ++ |
| $CoBr_2(H_2O)$(FFAAPTS) | 12 | 10 | 11 | 10 | ++ | ++ |
| $Co(NO_3)_2(H_2O)$(FFAAPTS) | 11 | 10 | 10 | 11 | ++ | ++ |
| $Co(NCS)_2(H_2O)$(FFAAPTS) | 15 | 14 | 16 | 15 | +++ | ++ |
| $Co(CH_3COO)_2(H_2O)$(FFAAPTS) | 11 | 10 | 12 | 10 | ++ | ++ |
| $Co(ClO_4)_2$(FFAAPTS)$_2$ | 11 | 10 | 11 | 11 | ++ | ++ |
| $CoCl_2(H_2O)$(4'$NO_2$ BAAPTS) | 12 | 10 | 11 | 12 | + | + |
| $CoBr_2(H_2O)$(4'-$NO_2$ BAAPTS) | 11 | 11 | 10 | 09 | + | + |
| $Co(NO_3)_2(H_2O)$(4'-$NO_2$ BAAPTS) | 10 | 09 | 11 | 10 | + | + |
| $Co(NCS)_2(H_2O)$(4'-$NO_2$ BAAPTS) | 12 | 11 | 10 | 08 | + | + |
| $Co(CH_3COO)_2(H_2O)$(4'-$NO_2$BAAPTS) | 10 | 09 | 11 | 10 | + | + |
| $Co(ClO_4)_2$(4'-$NO_2$BAAPTS)$_2$ | 11 | 10 | 09 | 10 | + | + |
| Ampicillin | 24 | 22 | 17 | 16 | - | - |
| Tetracycline | 18 | 17 | 21 | 22 | - | - |
| **Salicylic acid** | - | - | - | - | ++++ | ++++ |

### CONCLUSION

The type of complexes isolated during the present study demonstrate that interactions of $Co^{2+}$ and $Ni^{2+}$ salts with thiosemicarbazones lead to complexes with 1:1 and 1:2 stoichiometries. Co(II) complexes of both the thiosemicarbazones showed moderate antibacterial activities against *E.coli* and *S.typhi*. The compounds have antifungal activity against *A.niger* and *C.albicans*. FFAAPTS complexes showed increased antifungal activity than that of 4'-$NO_2$BAAPTS complexes. The overall experimental evidence shows that these metal ions display a coordination number six and presumably have a distorted octahedral environment around the metal ion as shown in Figures 1 and 2.

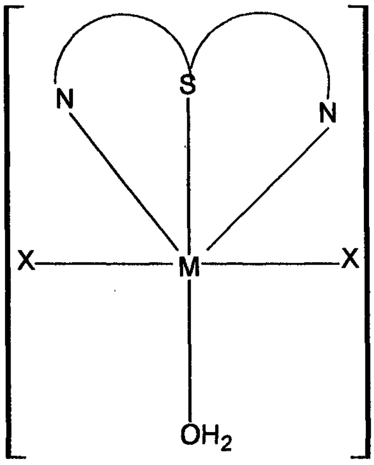

**Fig. 1:** Probable structure of Ni and Co metal complexes of thiosemicarbazone: $[MX_2(H_2O)(L)]$; (M = $Co^{2+}$ or $Ni^{2+}$; X = Cl, Br, $NO_3$ , NCS or OAc and L = FFAAPTS or 4'-$NO_2$BAAPTS).

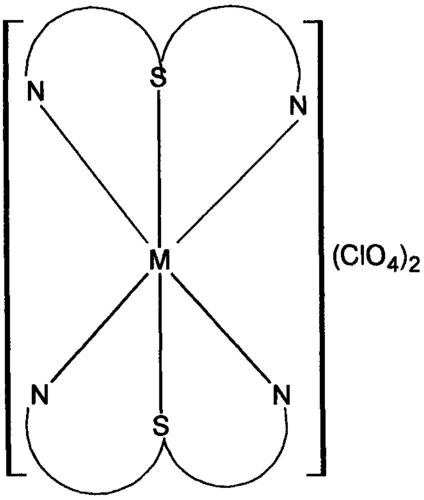

**Fig. 2:** Probable structure of Ni and Co metal complexes of thiosemicarbazone: $[M(ClO_4)_2(L)_2]$; (M = $Co^{2+}$ or $Ni^{2+}$ and L = FFAAPTS or 4'-$NO_2$BAAPTS).

### ACKNOWLEDGEMENT

The authors (RKA, SP) are grateful to the University of the South Pacific, Suva, Fiji, for the financial support in form of a research project (6395-1321) from the URC grant.

### REFERENCES

1. J. R. Wasson, G. M. Woltermann and H. J. Stockloosa, *Transition Metal Dithio- and Diselenophosphate Complexes in Inorganic Chemistry, Topics in Current Chemistry,* Springer Verlag, Berlin, **35,** 65 (1973).
2. G. D. Thoem and R. A. Ludwig, *The Dithiocarbamates and Related Compounds*, Elsevier, Amsterdam 1962.
3. D. Coucouvanis, *Prog. Inorg. Chem.*, **11,** 233 (1970).
4. C. K. Jorgensen, *Inorg. Chim. Acta Revs,* **2,** 65 (1968); J. A. McCleverty, *Prog. Inorg. Chem.,* **10,** 49 (1968).
5. S. E. Livingstone, *Coord. Chem. Rev.*, 7, 59 (1971); M. Cox and J. Darken, *Coord. Chem. Rev.*, 7, 29 (1971).
6. G. C. Pellaconi and T. Feltri, *Inorg. Nucl. Chem. Letters*, **8,** 325 (1972).
7. N. N. Orlova, V. A. Aksenova, D. A. Selidovkin, N. S. Bogdanova and G. N. Parshin, *Russ. Pharm. Toxic.*, 348 (1968).
8. K. Butler, U.S. Patent No. 3, 382, 266 (1968).
9. D. J. Baner, L. St. Vincent, C. H. Kempe and A. W. Douene, *Lancet,* **2,** 494 (1963).
10. H. G. Petering, H. H. Buskirk and G. E. Underwood, *Cancer Res.*, **64,** 367 (1964).
11. W. H. Wagner and E. Winkelmann, *Arzeim-Forsch,* **22,** 1713 (1972); R. Protivinsky, *Antibiot. Chemothes.* (Basal), **17,** 101 (1981).
12. A. Lewis and R. G. Shepherd, in A. Burger (Ed.), *Medicinal Chemistry*, Wiley, New York, p. 431 (1970).
13. P. Malatesta, G. P. Accinelli and G. Quaglia, *Ann. Chim.* (Rome), **49,** 397 (1959).
14. J. C. Logan, M. P. Cox, J. H. Morgan, A. M. Makohon and C. J. Pfan, *J. Gen. Virol.*, **28,** 271 (1975); C. Shipman (Jr.), S. H. Smith, J. C. Drach and D. L. Klaymann, *Antimicrol. Agents Chemother.*, **19,** 682 (1981).
15. A. Kamhiski, *Prensa Med. Agents*, **40,** 1263 (1953).
16. H. R. Wilson, G. R. Ravankar and R. L. Tolman, *J. Med. Chem.*, **17,** 760 (1974).
17. E. Winkelmann, W. H. Wagner and H. Wirth, *Arzeim Forsch*, **27,** 950 (1977).
18. D. L. Klayman, J. E. Bartosevich, T. S. Graffin, C. J. Mason and J. P. Scovill, *J. Med. Chem.*, **22,** 855 (1979); D. L. Klayman, J. P. Scovill, J. F. Bartosevich and C. J. Mason, *J. Med. Chem.*, **22,** 1367 (1979); D. L. Klaymann, J. P. Scovill, J. F. Bartosevich and C. J. Mason, *Eur. J. Med. Chem. (Theor.),* **16,** 317 (1981).
19. L. W. Johnson, J. W. Joyner and R. P. Perry, *Antibiotics and Chemotherapy,* **2,** 626 (1952).

20. H. W. Gausman, C. I. Rhykerd, H. R. Hinderliter, E. S. Scott and L. F. Audreith, *Botan. Gazz.*, **114**, 292 (1953); B. G. Benno, B. A. Gingras and C. H. Bayley, *Appl. Microbiol.*, **8**, 353 (1961).
21. K. Liebermeister, *Z. Naturforsch*, **85**, 79 (1950).
22. J. A. Crim and H. G. Petering, *Cancer Res.*, **27**, 1278 (1967); H. G. Petering and G. J. Van Giessman, *The Biochemistry of Copper*, Academic Press, N.Y. p. 197 (1966).
23. S. E. Livingstone, *Quart. Rev. Chem. Soc.*, **19**, 386 (1965).
24. M. A. Ali and S. E. Livingstone, *Coord. Chem. Revs.*, **13**, 101 (1974).
25. M. J. M. Campbell, *Coord. Chem. Rev.*, **15**, 279 (1975).
26. K. C. Agarwal and A. C. Sartorelli, in *Progress in Medicinal Chemistry*, G. P. Ellis and G. B. West (Eds.) Elsevier, **15**, 321 (1978).
27. S. Padhye and G. B. Kauffman, *Coord. Chem. Revs*, **63**, 127 (1985).
28. D. X. West, S. B. Padhye, P. B. Sonawane and R.C. Chikate, *Asian J. Chem. Revs.*, **1**, 125 (1990).
29. B. K. Rai and A. Baluni, *Asian J. Chem.*, **14**, 305 (2002).
30. B. K. Rai and P. Choudhary, *Asian J. Chem.*, **14**, 312 (2002).
31. J. Thomas and G. Parameswaran, *Asian J. Chem.*, **14**, 1354,1370 (2002).
32. N.T. Akinchan, P.M. Drozdzewski and R. Akinchan, *Polish J. Chem.*, **76**, 1381 (2002).
33. A. Sreekanth and M. R. P. Kurup, *Polyhedron*, **22**, 3321 (2003).
34. A. Sreekanth, S. Sivakumar and M. R. P. Kurup, *J. Mol. Struct.*, **655**, 47 (2003).
35. R. P. John, A. Sreekanth, M. R. P. Kurup, A. Usman, I. A. Razak and H. K. Fun, *Spectrochim. Acta*, **59A**, 1349 (2003).
36. A. Sreekanth and M. R. K. Kurup, *Polyhedron*, **23**, 969 (2004).
37. R. K. Agarwal, G. Singh and B. Bhushan, *J. Inst. Chem.(India)*. **65**, 131 (1993).
38. F. J. Welcher, *The Analytical Uses of Ethylenediamine Tetracetic Acid*, D. Van Nostrand Co. Inc., New York (1965).
39. A. I. Vogel, *A Text Book of Quantitative Inorganic Analysis*, 4th Edit., ELBS Longman, London (1986).
40. E. Kurz, G. Kober and M. Berl, *Anal. Chem.*, **30**, 1983 (1958).
41. W. J. Geary, *Coord. Chem. Rev.*, **7**, 110 (1971).
42. R. K. Agarwal and G. Singh, *Asian J. Chem.*, **1**, 409 (1989).
43. R. K. Agarwal, *Polish. J. Chem.*, **65**, 1211 (1991).
44. T. S. Lane, A. Yamagauchi and A. R. James, *J. Am. Chem. Soc.*, **80**, 527 (1958).
45. D. Banerjea and J. P. Singh, *Indian J. Chem.*, **6**, 34 (1968).
46. V. B. Rana, *J. Inorg. Nucl. Chem.*, **37**, 1826 (1975).
47. P. W. Sadler, *J. Chem. Soc.*, 957 (1961).
48. B. D. Sharma and J. C. Bailer (Jr.), *J. Am. Chem. Soc.*, **77**, 5476 (1955).
49. K. Nakamoto, *Infrared Spectra of Inorganic and Coordination Compounds*, Wiley, New York (1970).
50. J. R. Ferraro, *Low Frequency Vibrations of Inorganic and Coordination Compounds*, Plenum, New York (1971).
51. B. J. Hathaway and A. E. Underhill, *J. Chem. Soc.*, 3091 (1961).
52. S. D. Ross, *Spectrochim. Acta*, **18**, 225 (1962).

53. J. L. Burmeister, *Coord. Chem. Rev.*, **1**, 205 (1966), **3**, 225 (1968); **105**, 77 (1990).
54. C. C. Addison and N. Logan, *Quart. Chem. Rev.*, **25**, 289 (1971).
55. R. W. Hester and W.L. Grossman, *Inorg. Chem.*, **5**, 1308 (1966).
56. G. Topping, *Spectrochim. Acta*, **21**, 1743 (1956).
57. A. B. P. Lever, E. Mantiovani and B. S. Ramaswamy, *Can. J. Chem.*, **49**, 1957 (1971).
58. K. Nakamoto, J.Y. Morimoto and A.E. Martell, *J. Am. Chem. Soc.*, **83**, 4528 (1961).
59. I. S. Ahuja, C. L. Yadava and S. Tripathi, *Asian J. Chem.*, **1**, 195 (1989).
60. J. Reedijk, W. L. Driessen and W. L.Groenveld, *Recl. Trav. Chim.*, **88**, 1095 (1969).
61. A. B. P. Lever, J. Lewis and R.S. Nyholm, *J. Chem. Soc.*, 4751 (1964).
62. D. M. L. Goodgame and L. M. Venanzi, *J. Chem. Soc.*, 5909 (1963).
63. A. B. P. Lever, *Coord. Chem. Rev.*, **3**, 119 (1968).
64. N. Murthy and T. S. Dharamarajan, *Asian J. Chem.*, **14**, 1325 (2002).
65. M. R. Taylor, J. P. Glusker, E. J. Grabe and J. A. Minkins, *Bioinorg. Chem.*, **3**, 89 (1974).
66. M. B. Ferrari, G. G. Fava, P. Tarasconi, R. Albertini, S. Pineli and R. Starcich, *J. Inorg. Biochem.*, **53**,13 (1994).
67. M. B. Ferrari, G.G. Fava, E. Leporti, G. Pelosi, P. Tarasconi, R. Albertini, A. Bonati, P. Lunghi and S. Pineli, *J. Inorg. Biochem.*, **70**,145 (1998).

